# Needle-free injection of 5-aminolevulinic acid in photodynamic therapy for the treatment of condylomata acuminata

**DOI:** 10.3892/etm.2013.1092

**Published:** 2013-04-29

**Authors:** XIULI LI, XIUXIU WANG, JUNYING GU, YUE’E MA, ZHIYU LIU, YULING SHI

**Affiliations:** Department of Dermatology, Shanghai Tenth People’s Hospital, Tongji University School of Medicine, Shanghai 200072, P.R. China

**Keywords:** condylomata acuminata, 5-aminolevulinic acid, photodynamic therapy, needle-free injection

## Abstract

The external application of 5-aminolevulinic acid (ALA) in photodynamic therapy (PDT) results in a shallow penetration depth in thick or extensive condylomata acuminata (CA) lesions, thus demonstrating a poor therapeutic effect for those patients. To compare the efficacy of needle-free injection with external application of ALA in PDT for the treatment of CA, 160 CA patients with thick or extensive warts received ALA-PDT by means of external application or needle-free injection of ALA, respectively. The complete response (CR) rate and recurrence rate in the two groups were analyzed. The CR rate after the first treatment in the needle-free injection group (68.8%) was significantly higher compared with that in the external application group (52.5%; P=0.035). The recurrence rates in the needle-free injection group and external application group were 4.1 and 15.4%, respectively (P=0.022). The needle-free injection of ALA increases the therapeutic effect of PDT for CA patients with thick or extensive lesions. It shortens the treatment time and reduces the recurrence rate, and has great potential in the treatment of CA.

## Introduction

Condylomata acuminata (CA) is a common sexually transmitted disease (STD) caused by human papilloma virus (HPV) infection. The conventional treatments for CA include surgical excision and physical therapies, including cryotherapy, carbon dioxide laser treatment, electrofulguration and topical cytotoxic medication. However, the majority of these methods are traumatic and have local side-effects, including ulceration, infection and scar formation. More importantly, they are only used for visible warts and are invalid for subclinical or latent infection. This is one of the most important reasons why the treatment for CA is often associated with a very high recurrence rate (1).

The use of topical photodynamic therapy (PDT) with 5-aminolevulinic acid (ALA) has been explored in the treatment, as well as the diagnosis, of various proliferative skin diseases and cutaneous malignancies. It was first applied in dermatology in 1990 (2). Since then, ALA-PDT has shown its unique effect and has become increasingly widespread in clinical applications. Previously, ALA-PDT has been applied in the treatment of CA. Studies have shown that ALA-PDT is an effective technique for superficial anogenital warts and latent HPV infections. It is considered a simpler, more effective and safer therapy with a lower recurrence rate compared with conventional treatments (3–8). However, in clinical experience we identified that for patients with thick or extensive warts, a single ALA-PDT treatment does not completely eliminate the warts. Accordingly, a number of patients with such warts required one or more additional therapies to achieve a satisfactory result. We consider that this may be due to the limited penetration depth of ALA. A number of reports have indicated that the external application of ALA results in a shallow penetration depth of <2 mm into the tissue (9,10). Therefore, ALA is not able to sufficiently enter and destroy the affected lesions.

With the aim of improving the treatment, as well as alleviating the suffering of patients, we tested a new method of applying ALA to the topical lesions using needle-free injection. Needle-free injection has been applied in the medical field for over 70 years and the technology continues to be developed (11). However, it is rarely used in the dermatological field. We conducted a randomized-controlled trial to compare its efficacy and recurrence rate with those of conventional external application of ALA in PDT for the treatment of CA. Our study demonstrated a significant and novel method of ALA-PDT.

## Patients and methods

### 

#### Patients

From January 2012 to July 2012, 160 patients with CA were enrolled in the present study. All patients were outpatients from the Department of Dermatology at Shanghai Tenth People’s Hospital. The diagnosis of CA was made based on clinical symptoms, the acetowhitening test and HPV detection by polymerase chain reaction (PCR). All warts were located on the external genitals, distal urethra or perianal area with a thickness >3 mm. Patients were excluded if they had been treated with systemic immunotherapies within the preceding 4 weeks and/or external antiviral drugs within the preceding 2 weeks, had other STDs, were pregnant or were breast-feeding. Patients with intra-anal, intra-vaginal and/or cervical condylomata were also excluded from this study. All patients provided informed consent. The study protocol was reviewed and approved by the Ethics Committee of Shanghai Tenth People’s Hospital.

The eligible patients were randomly allocated into the external application group (n=80) or the needle-free injection group (n=80). In the external application group, 43 (53.8%) were males and 37 (46.2%) were females with an age range of 21–55 years (mean, 30.9±7.70 years). The duration of lesions was 53.90±13.63 days. The thickness of lesions varied from 3 to 12 mm (mean, 8.6±1.2 mm). Among the needle-free injection group, 44 (55.0%) were males and 36 (45.0%) were females with an age range of 22–49 years (mean, 35.9±6.10 years). The duration of lesions was 48.70±11.85 days. The thickness of lesions varied from 3 to 12 mm (mean, 7.8±0.9 mm).

#### Device

The photodynamic therapy apparatus (Wuhan Yage Laser Apparatus Co., Ltd., China) was able to emit 632.8 nm helium-neon laser light, with a power output of 100 mW and energy fluency of 100–150 J/cm^2^.

The INJEX device (Rösch AG Medizintechnik, Berlin, Germany) consisted of a spring-loaded variable-dose injector to which a disposable ampoule was attached. When activated, the trigger released the spring propelling a liquid medication with high velocity (140 m/sec) through a micro-orifice in the tip of the ampoule. The jet stream of medication traversed the skin and dispersed subcutaneously. The spring pressure and orifice diameter were designed for a penetration depth of 3–10 mm from the surface.

### ALA-PDT therapy

*Preparation of ALA.* ALA (Shanghai Fudan Zhangjiang Bio-Pharmaceutical Co., Ltd., China) was preserved in a −20°C freezer. The 20% ALA solution was freshly prepared each time before treatment by dissolving ALA in sterile 0.9% NaCl.

*Sterilization of the lesions.* Patients with genital lesions were asked to urinate prior to therapy. Hairs around the lesions were removed if necessary and 75% alcohol was used to sterilize the skin and mucosa around the lesions 2 or 3 times.

*External application group*. The 20% ALA solution was first applied on the warts and the adjacent normal skin (5 mm border). Then, a 20% ALA-soaked absorbent cotton ball was placed to cover the entire lesion area. Finally, the skin was covered with cling film and gauze for light protection. Patients were asked to lie down for 3 h. After 3 h, all the covers were removed and the mucosa was cleaned with sterile saline.

*Needle-free injection group.* The application of INJEX was performed in accordance with the manufacturer’s instructions. First, 0.4 ml 20% ALA solution was loaded into the ampoule and inserted into the injector. The injector was then held against the lesion. Upon actuation to release the spring, a rod rapidly pushed the syringe piston forward, pressurizing and ejecting the solution through the syringe orifice into the lesions and subcutaneous tissue. Patients were asked to lie down for 1.5 h.

*Irradiation.* Light irradiation of 100 J/cm^2^ at 100 mW was applied to the lesions and the adjacent normal skin (5 mm border) using the laser fiber for 20–40 min. Preparations were made to ensure the lesions were entirely covered by the laser light spot during the illumination.

The same treatment was repeated 7 days later if the warts were not completely removed.

#### Observation and evaluation

The lesions were evaluated before and one week after each ALA-PDT treatment by the same trained dermatologist. If the warts were not totally cleared after one week, a repeat treatment was administered. Follow-up evaluations for those patients whose warts were completely removed were conducted at 4, 8 and 12 weeks after the final treatment.

The indicators of efficacy were the complete remission (CR) and the recurrence rate. Patients were designated as in CR if all the lesions disappeared and the mucosa was restored to normal. A recurrence was defined as an occurrence of new lesions within 12 weeks in the same spot or within a 2 cm radius following CR. All patients were asked to report any adverse symptoms.

#### Statistical analysis

The recurrence rate and CR rate in the two groups were evaluated with the χ^2^ test. P<0.05 was considered to indicate a statistically significant difference. SPSS 17.0 software (SPSS, Inc., Chicago, IL, USA) was used to analyze the data.

## Results

The CR rate following the first treatment in the needle-free injection group (68.8%, 55/80) was significantly higher compared with that in the external application group (52.5%, 42/80; P=0.035; Table I). The total CR rate in the needle-free injection group following two treatment cycles (92.5%, 74/80) was also higher compared with that in the external application group (81.3%, 65/80; P=0.044; Table II).

During a follow-up study lasting 12 weeks, the recurrence rates in the needle-free injection group and external application group were 4.1% (3/74) and 15.4% (10/65), respectively, which were significantly different (P=0.027; Table III). One patient in the needle-free injection group dropped out during the follow-up; this case was considered as a recurrence.

## Discussion

Previous studies have indicated that ALA-PDT is an effective, safe and minimally invasive method for the treatment of superficial CA (3–8). PDT is a specialized form of photochemotherapy. There are three essential factors for effective PDT: oxygen, photosensitizer and light. The conventional ALA-PDT starts with the external application of ALA to the lesions. Over a period of time (∼3 h), ALA becomes concentrated in the lesions compared with the surrounding normal tissue (12). ALA is a natural precursor of heme (13). ALA itself is not a photosensitizer; however, after being applied topically, it is selectively absorbed by the rapidly proliferating cells that are infected by HPV and then transforms to endogenous protoporphyrin IX (PpIX) via biological synthesis. PpIX is an extremely powerful photosensitizer. At this point, a light of a certain wavelength (∼635nm) is used to illuminate the target tissue. PpIX is then activated by the irradiation, leading to the formation of singlet oxygen (^1^O_2_) by energy transfer, which results in cellular damage or destruction and eventually achieves the targeted therapeutic effect without harming the normal tissue. In addition to the direct killing effect, ALA-PDT may also have certain antiviral activities (14,15). The photosensitizer binds to virion surface glycoproteins and thus impedes the early steps of the infection cycle (16).

However, the efficacy of ALA-PDT is largely affected by the PpIX production level, which is limited by the penetration depth of ALA into the virus-effected lesions. Therefore, for patients with thick or extensive warts, it is often extremely difficult to produce a satisfactory result by external application of ALA in PDT. Patients often require a conventional treatment like curettage, cryotherapy or carbon-dioxide laser prior to ALA-PDT to first remove the visible warts. Yet those conventional treatments are often more painful and are associated with worse side-effects, including ulcers or scarring. Consequently, they have a higher recurrent risk and require a longer recovery time. In addition, the combined treatments not only prolong the treatment time, but also demand more costs; thus, they bring patients a lot of pressure, psychologically and economically.

For these reasons, the application of ALA intralesionally, as opposed to externally, has been suggested as a means to increase the penetration of the photosensitizers (17). There have been clinical studies on intralesional injection of ALA in PDT for the treatment of squamous cell carcinoma (SCC) (18), basal cell carcinoma (BCC) (19), Paget’s Disease (20) and other dermatoses (21). These studies presented favorable results. A previous study confirmed that local intracutaneous injection of ALA resulted in higher fluorescence and PpIX levels in contrast to the external application (22). However, the traditional needle injection also has its deficiencies. First of all, needles and syringes often become the object of fear and avoidance among patients with needle phobia. Even for normal patients, the pain caused by the needle injection is almost unavoidable and this often adversely affects the compliance of the therapy. As well as these drawbacks, the needle injection also has a higher chance of side-effects, including infection, vascular compromise, local necrosis and atrophy.

With all of these concerns in mind, we conducted the current study to identify a novel method of ALA administration, which enhances the penetration depth and also has minimal side-effects. As an alternative to needle injection, needle-free injection (also known as jet injection) may also enhance the delivery of ALA and distribute it more evenly without the side-effects produced by the use of needles. The needle-free injection was initially developed for mass inoculations around the late 1940s (23). In recent years, needle-free delivery technology has gained momentum in clinical areas and has been used for the delivery of not only high molecular weight compounds like insulin and growth hormones, but also local anesthetics and genes. Studies have shown that drugs delivered by needle-free injectors are generally absorbed into the body as quickly as those delivered by conventional subcutaneous needle injection (24). Also, the needle-free system results in a similar release from the deposit and reabsorption into the circulation compared with conventional needle administration (25).

Inspired by these experiences, we opted for needle-free injection of ALA as opposed to the conventional external application in the PDT treatment. The INJEX device used in this study was a needle-free delivery device approved by the Food and Drug Administration (FDA) for subcutaneous medications. The technology utilizes a spring mechanism to propel 0.03–0.5 ml solution from a single use disposable ampoule at an extremely high velocity (typically >100 m/sec) to pass through the skin into the subcutaneous tissue. This technique therefore obviates the risk of needle-based injections, including needle stick injuries (NSIs) and cross-contamination. It also addresses the pain and needle-phobia caused by needle injection. Furthermore, our study demonstrated that the interval time prior to the irradiation may be much shorter (1.5 h) with the needle-free injector than with the conventional method (3 h), which indicates that the onset of therapeutic effects following administration using the needle-free injector is much faster than using external application. The same conclusion was obtained by Inoue *et al* (26) who compared the needle-free injector with transdermal patches.

In the current study, the needle-free injection of ALA in PDT demonstrated a satisfactory therapeutic effect and low recurrence rate compared with the conventional ALA-PDT for CA patients with thick or extensive warts. The CR rate following the first treatment was significantly higher compared to the external application group. The total CR rate following two treatment cycles was also significantly higher than that in the external application group. The recurrence rate was much lower than that in the external application group. No specific side-effects were observed in the two groups.

The results of this study are extremely encouraging. We demonstrated that with needle-free injection, patients were able to achieve more satisfactory therapeutic effects than with the conventional external application of ALA in PDT. This presents a potential method of treating CA with reduced treatment time and lower recurrences. Particularly for patients with thick or extensive warts, we consider that the needle-free technique has strong advantages over the conventional methods. However, supplementary studies with larger sample numbers, as well as a longer follow-up, are required for further evaluation of this promising technique. With more clinical applications and experiments, other types of dermatoses, including BCC, SCC and Bowen’s disease, may also benefit from this novel method.

ALA-PDT is an effective, minimally invasive and easily replicable treatment for CA. The present study demonstrated that needle-free injection of ALA in PDT is an excellent and satisfactory therapeutic method in treating CA patients with thick or extensive warts.

It is suggested that needle-free injection of ALA in PDT has the potential to lead to significant advances in the treatment of CA, including fewer side-effects, shorter treatment cycles, faster treatment time, improved compliance and reduced recurrence rate.

## Figures and Tables

**Table I.** Complete remission (CR) rate in the two groups following the first treatment.

Group	Total cases	CR (n)	CR rate (%)	χ^2^	P-value
Needle-free injection	80	55	68.8	4.425	0.035
External application	80	42	52.5		

**Table II.** Total complete remission (CR) rate in the two groups following two treatment cycles.

Group	Total cases	CR (n)	CR rate (%)	χ^2^	P-value
Needle-free injection	80	74	92.5	4.066	0.044
External application	80	65	81.3		

**Table III.** Recurrence rate in the two groups over the 12-week follow-up.

Group	Cure number	No. of recurrences (4, 8, 12 weeks)	Recurrence rate (%)	χ^2^	P-value
Needle-free injection	74	3 (2, 1, 0)	4.1	5.240	0.022
External application	65	10 (6, 3, 1)	15.4		
